# Assessment of quality in psychiatric nursing documentation – a clinical audit

**DOI:** 10.1186/1472-6955-13-32

**Published:** 2014-10-17

**Authors:** Marit Helen Instefjord, Katrine Aasekjær, Birgitte Espehaug, Birgitte Graverholt

**Affiliations:** 1 Helse More og Romsdal Health Trust, Ålesund, Norway; 2 Centre for Evidence-Based Practice, Bergen University College, Bergen, Norway

**Keywords:** Clinical audit, Evidence-based nursing, Nursing documentation, Nursing process, Psychiatric nursing

## Abstract

**Background:**

Quality in nursing documentation facilitates continuity of care and patient safety. Lack of communication between healthcare providers is associated with errors and adverse events. Shortcomings are identified in nursing documentation in several clinical specialties, but very little is known about the quality of how nurses document in the field of psychiatry. Therefore, the aim of this study was to assess the quality of the written nursing documentation in a psychiatric hospital.

**Method:**

A cross-sectional, retrospective patient record review was conducted using the N-Catch audit instrument. In 2011 the nursing documentation from 21 persons admitted to a psychiatric department from September to December 2010 was assessed. The N-Catch instrument was used to audit the record structure, admission notes, nursing care plans, progress and outcome reports, discharge notes and information about the patients’ personal details. The items of N-Catch were scored for quantity and/or quality (0–3 points).

**Results:**

The item ‘quantity of progress and evaluation notes’ had the lowest score: in 86% of the records progress and outcome were evaluated only sporadically. The items ‘the patients’ personal details’ and ‘quantity of record structure’ had the highest scores: respectively 100% and 71% of the records achieved the highest score of these items.

**Conclusions:**

Deficiencies in nursing documentation identified in other clinical specialties also apply to the clinical field of psychiatry. The quality of electronic written nursing documentation in psychiatric nursing needs improvements to ensure continuity and patient safety. This study shows the importance of the existence of a validated tool, readily available to assess local levels of nursing documentation quality.

## Background

High quality nursing documentation aims to promote structured, consistent and effective communication between caregivers, and facilitate continuity and patient safety [1-4]. This links nursing documentation directly to the internationally recognized quality and patient safety aspects of health care [5]. Inadequate communication between caregivers is associated with discontinuity of care, a factor that contributes to errors [6-8]. The consequences of discontinuity of care are linked to increased cost and length of hospital stay, readmissions, poorer patient satisfaction, adverse events, delays in treatment and diagnosis, inappropriate treatment and omission of care [9,10].

Systematic reviews covering several clinical specialties such as postoperative care, wound care, orthopedic care, pain management and nursing homes, have shown deficiencies in the quality of nursing documentation. Saranto & Kinnunen [1] found that the recorded patient data were partly inaccurate, inadequate and lacked important details of the nursing process. Müller-Staub *et al*. [11] reported shortcomings in the nursing diagnostic process where documentation of signs, symptoms and etiology was found to be insufficient. Wang *et al.*[4] found incoherence in documentation of the nursing process, indicating that nursing care was not sufficiently documented in the records, and that written communication concerning the patient between caregivers was inadequate. In a qualitative metasynthesis, Kärkkäinen *et al.*[12] found that the amount of documentation assigned to patients’ own requests and needs was small. Further, these reviews of the literature generally find shortcomings in the nursing care plans, and little evidence of patient involvement in defining diagnoses and goals. Although there is no reason to believe that the quality of nursing documentation is better in the clinical field of psychiatry, this appears to lack focus in the literature.

As such, the aim of this study was to assess the quality of electronic written nursing documentation in psychiatric patient records against research recommendations for best practice.

## Methods

Clinical audit was used as a stepwise quality improvement process through a systematic review of care against explicit criteria [13]. To review the nursing documentation, a cross-sectional, retrospective patient record review was conducted using the N-Catch audit instrument [14].

### Setting

Psychiatric care in Norway is organized at hospital and community level. At hospital level, different departments treat patients according to severity of symptoms. The patients are transferred from acute inpatient departments to sub-acute departments as their condition improves. This study took place in a sub-acute psychiatric hospital department. The department had 16 beds in two wards, and accepts patients aged 18 years or older with mental health problems such as psychosis, depressions and anxiety disorders. The average length of stay at the department in 2010 was 27 days.

All nurses (n = 13) in the study setting had completed a one-day course in nursing documentation in electronic patient records. Most of them (85%) had a degree in psychiatric nursing, and at least 10 years of relevant experience.

The nursing section of the electronic patient record is structured using the VIPS model, a validated model designed to structure nursing documentation systematically according to the nursing process [15]. VIPS is an acronym for the Swedish terms for well-being, integrity, prevention and safety. These terms are seen as major goals for nursing care [16].

### Sample

In most audits, a time interval of one to three months is sufficient [17]. A three month time interval was chosen to assess electronic written nursing documentation (1^st^ September – 1^st^ December 2010). The interval was chosen as it represents a stable period of regular clinical activity, with no public holidays interfering with activities. Statistical power calculations showed that 20 patient records were needed to estimate the proportion that complied with the defined criteria, with a desired level of confidence of 95% and precision (margin of error) of 5% (http://www.raosoft.com/samplesize.html). We included the records of all patients admitted in this period (n = 21). If a patient was admitted several times, all admissions were included.

### Audit instrument

The N-catch audit instrument was used in this study. This is a Norwegian version of the D-Catch instrument, a valid and reliable measurement instrument developed in the Netherlands to assess nursing documentation in general hospital settings [18]. The N-Catch is culturally adapted from the D-Catch, and translated with a forward and backward translation according to scientific criteria [14,19]. N-Catch is designed to assess electronic written nursing documentation [14].

The N-Catch consists of eight items, and covers quantity and/or quality of the stages of the nursing process (Table 1): Assessment, nursing care plan with nursing diagnoses, outcomes and interventions, and evaluation in progress- and discharge notes. The different stages are scored on a scale from 0 – 3 points, where 3 points represent the highest score. The patients’ personal details are scored on a scale from 0–2 points.

**Table 1:** The criteria and standards of the items of N-Catch, the evidence base and sources of data collection

	**Criteria and standard (N-Catch)**	**Evidence base**	**Data source**
**The nursing process**	**The nursing record should contain:**	Wang, Hailey & Yu [4], Saranto & Kinnunen [1], The Norwegian Board of Health [20]	Nursing documentation in the nursing record
	• Assessment on admission		
	• The patient’s personal details		
	• Nursing care plan		
	• Progress and evaluation notes		
	**Standard:** 100% of the nursing records should fulfill these criteria		
	**The patient’s personal** details should include name, address, date of birth, telephone number, marital status, next of kin, occupation, general practitioner	The Norwegian Board of Health [20]	The personal details file in the patient record
	**Standard:** 100% of the patient records should contain these elements		
**Assessment**	**The assessment on admission:**	Wang, Hailey & Yu [4], Müller-Staub *et al*. [11], The Norwegian Board of Health [20]	The admission note in the nursing record
	• Quantity: The patient’s health history, the reason for admission and the patient’s health status should be completely documented		
	• Quality: The language should be correct and concise, and all relevant information should be included		
	**Standard:** 100% of the admission notes should fulfill these criteria		
**Nursing care plan Implementation**	**The nursing care plan:**	Wang, Hailey & Yu [4], Jefferies, Johnson & Griffiths [3], Suhonen, Välimäki & Leino-Kilpi [22], Ehnfors, Ehrenberg & Thorell-Ekstrand [16], The Norwegian Board of Health [20]	The nursing care plan in the nursing record
	• Quantity: The nursing care plan should be updated and individualized, and contain nursing diagnoses, nursing outcomes and nursing interventions.		
	• Nursing diagnoses: Should contain information about the symptoms, consequences and the patients’ resources		
	• Nursing outcomes: Should be measurable and realistic and describe a desired situation for the patient in the future		
	• Nursing interventions: Should be specific and linked to nursing diagnoses and nursing outcomes		
	**Standard:** 100% of the nursing care plans should fulfill these criteria		
**Evaluation**	**Evaluation - progress notes:**	Wang, Hailey & Yu [4], Jefferies, Johnson & Griffiths [3], Saranto & Kinnunen [1], Ehnfors, Ehrenberg & Thorell-Ekstand [16], The Norwegian Board of Health [20]	Progress and evaluation notes in the nursing record
	• Quantity: Progress reports should be written after each shift		
	• Quality: Progress reports should assess the patients’ health status according to nursing outcomes. The language should be correct and concise		
	**Standard:** 100% of the progress reports should fulfill these criteria		
**Evaluation**	**Evaluation - discharge notes:**	Wang, Hailey & Yu [4], Saranto & Kinnunen [1], The Norwegian Board of Health [20]	Discharge notes in the nursing record
	• Should contain relevant information important to understand the patients’ health status on discharge.		
	• Should contain evaluation of results.		
	• The language should be correct and concise.		
	**Standard:** 100% of the discharge notes should fulfill these criteria		

The first column adds how the nursing process relates.

We used requirements from Norwegian legislation to set the standard for degree of compliance with criteria. Accordingly, 100% of the nursing records should comply with the criteria for 3 (2) points [20]. All items of the N-Catch, except one, were scored. The excluded item relates to legibility of the entries and is not applicable to electronic records. Two of the items were adapted to the local setting: The first was the patients’ personal details. This item is not specific neither in the original D-catch instrument, nor the N-Catch instrument, but is a legal requirement in Norway (name, address, telephone number, date of birth, next of kin, marital state, occupation and general practitioner) [20]. The other item adapted concerned the nursing diagnoses. This was adapted to fit the local electronic documentation system (DocuLive), which requires that nursing diagnoses should hold information about symptoms, consequences and the patients’ resources. The modification was done to meet this requirement.

An inter-rater reliability test, using Cohen’s weighted kappa (κw), was calculated on the initial, independent scores of the entire sample. The results from the inter-rater reliability test varied from very good to fair (Table 2).

**Table 2:** Cohen’s weighted kappa (κw) inter-rater reliability of the N-Catch instrument

**Elements of N-Catch**	**Weighted kappa (κw)**
Which elements of the nursing record are present?	1.00
Admission note – quantity	0.39
Admission note – quality	0.42
Nursing care plan – quantity	0.60
Nursing diagnoses – quality	0.59
Nursing outcomes – quality	0.69
Nursing interventions – quality	0.23
Progress and evaluation notes – quantity	0.67
Progress and evaluation notes – quality	0.29
Discharge note – quality	0.47

### Data collection

Retrieval of the nursing documentation from the electronic patient record was done by the department secretary, who copied it, scored the patients’ personal details and then removed personal identifying information before handing it over to the reviewers.

Two reviewers, both nurses from the department, independently assessed the patient records against the criteria in the N-Catch. Prior to the assessment, the two reviewers studied the instrument and reached a common understanding of the items. Subsequent to the data collection, scores were compared and discussed until consensus was reached.

### Data analysis

For statistical analysis the Microsoft Office Excel 2007 and Vassar Stats online statistics software were used. Frequencies, percentage scores and means with confidence intervals for all the items in the N-Catch instrument were calculated.

### Ethical considerations

The local Data Protection Official for Research at Ålesund Hospital, The Møre and Romsdal Health Trust, reviewed the study and granted approval. The study was exempted from patient consent as only anonymous data was used. Prior to the investigation, the nurses at the study department were informed about the study methods and aims.

## Results

A total of 21 patient records from two psychiatric wards were examined according to the criteria in the N-Catch instrument (Table 3).

**Table 3:** The N-Catch scores of accuracy of documentation in the psychiatric nursing record

**Element of the N-Catch**	**Scale**		**Score, mean (95% CI)**	**N**	**Score 3 n (%)**	**Score 2 n (%)**	**Score 1 n (%)**	**Score 0 n (%)**
	**Quantity**	**Quality**						
Record structure	0 – 3		2.7 (2.54 – 2.89)	21	15 (71)	6 (29)	0	0
The patient’s personal details	0 – 2		2.0	21	0	21(100)	0	0
Admission note	0 – 3		0.9 (0.45 – 1.35)	20^1^	2 (10)	4 (20)	4 (20)	10 (50)
Admission note		0 - 3	1.3 (0.86 – 1.64)	20^1^	1 (5)	8 (40)	6 (30)	5(25)
Nursing care plan	0 – 3		1.7 (0.78 – 2.14)	21	6 (28,5)	6 (28,5)	5 (24)	4 (19)
Nursing diagnoses		0 - 3	1.5 (1.01 – 1.95)	21	4 (19)	3 (38)	3 (14)	6 (29)
Nursing outcomes		0 - 3	0.8 (0.29 – 1.23)	21	3 (14)	2 (10)	3 (14)	13 (62)
Nursing interventions		0 - 3	1.2 (0.86 – 1.52)	21	5 (14)	6 (28)	10 (48)	4 (19)
Progress report	0 – 3		0.2 (−0.02 – 0.42)	21	0	1 (5)	2 (9)	18 (86)
Progress report		0 - 3	1.4 (1.11 – 1.66)	21	0	10 (48)	9 (43)	2 (9)
Discharge note		0 - 3	1.7 (1.24 – 2.10)	18^2^	3 (16,7)	8 (50)	3 (16,5)	3(16,7)

^1^1/21 records had no admission note. The N-Catch had no scoring options for this eventuality.

^2^3/21 records had no discharge note. The N-Catch had no scoring options for this eventuality.

The highest accuracy scores were found in the documentation of personal details, with 100% of the records in accordance with criteria (mean score 2.0). Documentation of all the stages of the nursing process was found in 71% of the records, and in the remaining 29% all stages but one were documented. The mean score for this item was 2.7 points (CI 2.54 – 2.89).

To achieve the highest score for quantity of the admission report, it needed to include reason for admission, mental health history and patient health status on admission. Ten per cent of the admission reports reached full score and 50% of the reports held only one of these three parts. The mean score for this item was 0.9 points (CI 0.45 – 1.35).

Deficiencies were found in the nursing care plan as well. Merely 19% of the nursing diagnoses were correctly formulated, with symptoms, consequences and patients’ resources. Overall, this item had a mean score of 1.5 points (CI 1.01 – 1.95). Descriptions of outcomes were missing or inadequate according to criteria in 62% of the nursing care plans. This item had a mean score of 0.8 (CI 0.29 – 1.23). Only 5% of the nursing interventions were formulated in a way that specified the content and frequency of the intervention. The mean score was 1.2 points (CI 0.86 – 1.52) for this item.

The lowest scores of the investigation were found in the quantity of progress evaluation. No records achieved full score with progress reports after each shift, and 86% scored 0 points, implying that progress reports were written only sporadically. The mean score was 0.2 points for progress evaluation (CI −0.02 – 0.42).

Merely 17% of the discharge notes contained relevant information necessary to understand the patients’ health status and achieved outcomes at discharge or transfer, and three records lacked a discharge note. In 50% of the records, the discharge note provided relevant, but deficient information to the next care level. This item had a mean score of 1.7 (CI 1.24 – 2.10).

## Discussion

The findings from this audit suggest that electronic written nursing records only partly are in accordance with research recommendations for best practice, and that shortcomings of nursing documentation found in other clinical specialties apply to mental health nursing, as well. We assessed the quality and the quantity of electronic written nursing documentation, according to the N-catch audit instrument. We found that the recommended stages are generally present, with 71% of the records meeting the standard. The quality of the content, however, rates poorly. Only the criterion concerning the documentation of the *patients’ personal details* met the standard for the audit. *Admission reports* were only complete in 10% of the patient journals. In the *nursing care plans* diagnoses were formulated correctly in 19%, interventions in 5% and outcomes in 14% of the cases. *Progress notes* scored similarly low, with no records achieving full score. More specifically, 86% of the records scored zero, implying only sporadic documentation of this stage. *Discharge notes* were present in 86% of the records, but 40% of the discharge notes provided inadequate information to the next care level.

Several studies have found inadequate nursing documentation of *admission reports*[4,21]. Serious deficiencies in the admission reports have been found to cause shortcomings in the subsequent stages of the nursing documentation, emphasizing its importance throughout the continuum of documentation [21]. We found that only 10 % of the records were in line with the criteria, which perhaps is the most serious flaw in the audit.

The *nursing care plan* should accurately reflect the patients’ individual needs, and include diagnoses, interventions and outcomes. Individualized care has previously been shown to have positive effects on the quality of care by promoting wellness and health functioning in populations and by maintaining functional abilities and autonomy [22]. Nursing diagnoses should include symptoms, consequences and patient resources, according to the recommendations [11,20]. On the contrary, several studies have shown that nursing diagnoses often are limited to diagnostic labels [4,11,23]. This is the case for our investigation as well, where only one in five records fulfilled the criteria, which is insufficient to capture the patients’ needs in this hospital ward. Nursing interventions were only specific in content and frequency in 5% of the records we assessed. Such inadequate descriptions have been found in other studies too [24,25], indicating that nursing-specific interventions are not emphasized in the documentation. This may in turn create misunderstandings, discontinuity of care and as such compromise patient safety. A description of outcomes lacked in 62% of the records investigated, which is similar to what Stokke & Kalfoss [26] found. Without defined outcomes, nursing is fragmented, coincidental, non-individualized and ineffective.

The content of the nursing documentation should be continually revised [3,20]. *Progress notes* should evaluate the items in the nursing care plan to capture changes in the patients’ condition and ensure continuity of care [1,16,20,25]. In our assessment, progress notes were written sporadically in the majority of the nursing records. Previous studies have shown inconsistent results regarding the progress notes. Some studies have found excessive use of progress notes with little essence, obscuring vital information about the patient [26,27], while others have found results similar to our study with a scarcity of progress notes [25]. Nurses operate on a line of continuum here: On the one hand, nurses should document information that is essential to the patient cared for. But the commonly acknowledged idea that “if it is not documented, it is not done” obviously has a strong stand among nurses and may result in too detailed documentation with huge amounts of progress notes [28]. Linking progress notes to a structured nursing care plan can contribute to focused and effective communication between health professionals [11].

The *discharge note* should provide essential information about the patient to the next care level, and thus ensure continuity [20]. Previous research have found that discharge notes often are limited and deficient, causing communication problems between primary and secondary care settings [29]. Further, McKenna *et al.*[30] found that a majority of district nurses were dissatisfied with the content in the discharge notes from the hospitals. In our investigation, some of the best results were found in the discharge note. Only 16% obtained a full score, but 50% scored 2 points, indicating that nurses are aware of the importance of providing quality information about the patient to the next care level. However, 14% of the records lacked a discharge note, and in 33% of the discharge notes the information provided was inaccurate and inadequate. Inadequate information to the next level of care may compromise continuity and patient safety.

Although shortcomings were identified in almost all areas of the nursing records, some of these seem easier to improve than others. For instance, only 16% of the records obtained a full score in the discharge note, but half of the records scored 2 points for this item, and with little effort this could be improved. The same opportunity applies to the quality of the admission note and the quality of the progress notes.

The assessment was conducted on electronic written nursing documentation. This may have influenced the findings. We found that the description of nursing diagnoses, interventions and outcomes in the nursing care plans were inadequate. Previous research has found that the nursing care plans in electronic nursing records tend to be longer and less specific than those in manual records [4]. We also found that progress notes were written only sporadically. Rykkje *et al.*[31] found that nurses documented less after the introduction of electronic patient records. Since all documentation in the patient health record in Norway is electronic and we thus had no comparison, it is not possible to be certain that these findings are related to the nursing records being electronic.

### Limitations

The sample size in this study was small but adequate, as a time interval of one to three months is sufficient in most audits [17]. Further, measures were made to ensure a representative sample by choosing an interval which appeared the most stable period of regular activity in the department.

The Norwegian version of the audit instrument is not fully validated. Still, the psychometric properties of the Dutch version are established [18], and the translation of the instrument is undertaken according to scientific criteria [14,19].

A full audit cycle involves the implementation of improvement measures and a re-audit [32]. The implementation of improvement measures is not included in this study; however, the study is useful in the sense that it clearly reveals which areas of the documentation are in need of improvement.

The results from the inter-rater reliability test varied from fair to very good (κw 0.23 – 1.00). The differences in agreement may imply that the description for the scoring of the items is too vague and subject to interpretation. Reliability and validity of the N-Catch instrument should be tested more extensively before applied broader.

## Conclusion

In a psychiatric hospital department, we found that nurses only to a limited degree document patient care according to recommendations and legal requirements. This implies that deficiencies in nursing documentation identified in other clinical specialties also apply to the clinical field of psychiatry. This study shows the importance of the existence of a validated tool, readily available to assess local levels of nursing documentation quality. The N-Catch instrument was found useful in identifying areas where quality improvement is required in psychiatric nursing documentation.

## Competing interests

The authors declare that they have no competing interests.

## Authors’ contributions

MHI, KAA & BG are responsible for the study conception and design. MHI performed the data collection and the data analysis, and drafted the manuscript. KAA and BG made critical revisions to the paper. BE provided statistical expertise. All authors read and approved the final manuscript.

## Pre-publication history

The pre-publication history for this paper can be accessed here:

http://www.biomedcentral.com/1472-6955/13/32/prepub
